# Blood-Pressure-Lowering and Endothelium-Dependent Vasorelaxant Effects of Nutgall Tree in Rats

**DOI:** 10.3390/foods13071041

**Published:** 2024-03-28

**Authors:** Sujin Shin, Junkyu Park, Ho-Young Choi, Youngmin Bu, Kyungjin Lee

**Affiliations:** 1 Department of Korean Medicine, Graduate School, Kyung Hee University, Seoul 02447, Republic of Korea; sjshin04@khu.ac.kr; 2 Department of Science in Korean Medicine, Graduate School, Kyung Hee University, Seoul 02447, Republic of Korea; ojeoksan@khu.ac.kr; 3 Department of Herbal Pharmacology, College of Korean Medicine, Kyung Hee University, Seoul 02447, Republic of Korea; hychoi@khu.ac.kr (H.-Y.C.); ymbu@khu.ac.kr (Y.B.)

**Keywords:** nutgall tree, *Rhus chinensis*, blood pressure, hypertension, hypotensive effect, vasorelaxant, endothelium, NO/cGMP pathway, angiotensin II

## Abstract

Hypertension is the crucial modifiable risk factor for cardiovascular diseases, and efforts to identify functional foods that are effective for hypertension control are increasing. The nutgall tree (NT, *Rhus chinensis* Mill.) is used in traditional medicine and food because of its medicinal value. However, the role of NT in hypertension has not been investigated. Therefore, the hypotensive effect of NT leaf ethanol extract (NTE) was investigated in spontaneously hypertensive rats (SHRs). SHRs were allocated to three groups (control, 300, or 1000 mg/kg NTE), and blood pressure was measured before and after oral administration. Systolic and diastolic blood pressure significantly decreased in the NTE 1000 mg/kg group and was the lowest at 2 h after administration (−26.4 ± 10.3, −33.5 ± 9.8%, respectively). Daily NTE administration for five days also resulted in a similar effect. Further, the vasorelaxant effects and related mechanisms were investigated in the aortas of Sprague Dawley rats. NTE showed the dose-dependent blood-vessel-relaxing effect, and its mechanism involves the NO-sGC-cGMP pathway, activation of K^+^ channels, and reduction in the vasoconstrictive action of angiotensin II. Therefore, our study provides basic data indicating the potential use of NTE as a functional food for high blood pressure.

## 1. Introduction

Cardiovascular diseases (CVDs) are abnormalities of the heart and circulatory system that are the leading causes of premature death [1]. Hypertension is an important risk factor for CVDs, and the risk increases with earlier onset [2]. Maintaining blood pressure within an appropriate range of systolic blood pressure (SBP) < 140 mmHg and diastolic blood pressure (DBP) < 90 mmHg is crucial; however, the proportion of well-controlled blood pressure among hypertensive patients is only 13.8% [3,4]. A 5 mmHg decrease in SBP lowers the risk of CVDs, even in people with normal blood pressure [5]. Therefore, interest in and efforts to identify functional foods that are effective in controlling blood pressure are increasing [6].

Functional foods are novel foods that are safe and can help promote health and prevent diseases [7]. Plant extracts containing biologically active compounds are a good source of functional foods [8]. Plant extracts and their natural bioactive compounds have demonstrated beneficial effects in CVDs, such as lowering blood pressure and preventing endothelial dysfunction [9,10]. Polyphenols found in plants have been shown to regulate vascular tone through various pathways such as modulating nitric oxide (NO) production, increasing cyclic guanosine monophosphate (cGMP), activating K^+^ channels, or inhibiting Ca^2+^ channels [11].

Nutgall tree (NT), also known as the Chinese sumac or *Rhus chinensis* Mill, is distributed in the wide range of temperate, subtropical, and tropical regions [12]. Owing to their abundant nutrients, the leaf and fruit of NT have been used as ingredients in appetizers, spices, vinegar, drinks, and oils [13]. More importantly, almost all parts of NT, including the leaf, stem, root, fruit, seed, and *Galla chinensis*, have been used for the treatment of diarrhea, diabetes, jaundice, hepatitis, arthralgia, chronic cough, and cold [13]. Recently, a large number of studies have drawn attention to the medicinal value of NT, revealing a variety of bioactive compounds, including flavonoids, lignans, phenolic acids, tannins, fatty acids, and urushiols, and its biological activities include anti-oxidant, anti-viral, anti-diabetic, anti-obesity, anti-cancer, and hepatoprotective activities [14,15]. However, no studies have focused on the role of NT in hypertension.

The leaves of NT have a variety of polyphenolic compounds and are also rich in protein, vitamins, and trace elements, and efforts have been made to develop these leaves into functional beverages that have a detoxification effect and promote metabolism [15]. Therefore, the leaves of NT were extracted with 50% ethanol and were used to investigate their potential for treating hypertension. The hypotensive effect of NT leaf ethanol extract (NTE) was studied in spontaneously hypertensive rats (SHRs). In addition, its blood-vessel-relaxing effect was investigated using the Sprague Dawley (SD) rat aorta.

## 2. Materials and Methods

### 2.1. Plant Materials

NT was collected from Giheung-gu, Yongin-si, Gyeonggi-do, Republic of Korea in October 2021. This species was identified by Professor Weon-Ki Paik (Department of Life Science, Daejin University, Republic of Korea) and Kang-Hyup Lee (Korea National Arboretum, Republic of Korea). A voucher specimen was deposited at the College of Korean Medicine, Kyung Hee University, Seoul, Republic of Korea. The dried leaves of NT (100 g) were extracted by boiling in 1000 mL of 50% ethanol at 70 °C for 2 h and were filtered. The filtrate was concentrated by evaporator, and freeze-dried (yield: 17.25%).

### 2.2. Chemicals and Solution

Dimethyl sulfoxide (DMSO) was obtained from Junsei (Tokyo, Japan). N^G^-nitro-L-arginine methyl ester (L-NAME), 4-aminopyridine (4-AP), tetraethylammonium (TEA), and glibenclamide were obtained from Wako Pure Chemical Industries (Osaka, Japan). 1H-[1,2,4]oxadiazolo[4,3-a]quinoxalin-1-one (ODQ) was obtained from Tokyo Chemical Industry (Tokyo, Japan). Krebs–Henseleit (KH) solution used in this study was composed of 118.0 mM NaCl, 4.7 mM KCl, 2.5 mM CaCl_2_, 1.2 mM MgSO_4_, 1.2 mM KH_2_PO_4_, 11.1 mM D-glucose, and 25.0 mM NaHCO_3_. All the reagents used in KH solution, BaCl_2_, and urethane were obtained from Daejeong Chemical and Gold (Siheung-si, Republic of Korea). All other reagents were purchased from Sigma Aldrich (St. Louis, MA, USA).

### 2.3. Total Polyphenol Content

A total of 200 μg of freeze-dried NTE was dissolved in 20 mL of 80% ethanol. Total phenolic content was quantified via the Folin–Ciocalteu method [16]. Briefly, 200 μL of dissolved NTE was mixed with 200 μL of Folin–Ciocalteu reagent and 2 mL of 7.5% Na_2_CO_3_. The spectrophotometer (Epoch 2, BioTek Instruments Inc., Charlotte, VT, USA) was set to a wavelength of 750 nm. The total phenolic content of NTE was determined on the basis of a standard curve of the gallic acid standard. Data were expressed as mg gallic acid equivalent per gram of dried NTE weight.

Total flavonoid content was quantified according to the aluminum trichloride method [17]. A total of 500 μL of NTE was added to 150 μL of 5% NaNO_2_, 150 μL of 10% AlCl_3_, and 1 mL of 1 M NaOH. The spectrophotometer was set to a wavelength of 510 nm. The total phenolic content was determined on the basis of a standard curve of the catechin standard. Data were expressed as mg catechin equivalent per gram of dried NTE weight.

The total anthocyanin content was quantified by the pH differential method [18]. A total of 200 μL of NTE was added to 800 μL of 25 mM KCl buffer (pH 1.0) or 400 mM sodium acetate buffer (pH 4.5). The spectrophotometer was set to a wavelength of 520 and 700 nm. The total anthocyanin content was determined on the basis of cyanidin-3-glucoside. Data were expressed as mg cyanidin-3-glucoside equivalent per gram of dried NTE weight. The total polyphenol content of NTE is shown in Table 1.

### 2.4. Animals and Ethics Statement

Eighteen-week-old male SHR (SLC Inc., Shizuoka, Japan) were used to measure the blood-pressure-lowering effect. Six-week-old male SD rats (Daehan Biolink, Eumseong-gun, Republic of Korea) were used to investigate the vasorelaxant effects. Animals were maintained in cages under a 12/12 h light/dark cycle at a temperature of 22 ± 2 °C and humidity of 45–65%. The rats had unlimited access to the diet and tap water. All experiments were performed in accordance with the Animal Welfare Guidelines and approved by the Animal Experiment Ethics Committee of Kyung Hee University (KHSASP-23-506).

### 2.5. Measurement of the Acute Blood-Pressure-Lowering Effect

The SHRs were acclimated for one week prior to the experiment, and blood pressure was measured with the CODA 8-Channel High Throughput Non-Invasive Blood Pressure System (Kent Scientific Co., Torrington, CT, USA) once a day during the acclimation period for adaptation. The SHRs were allocated to three groups of six rats each: control group (1 mL distilled water orally), low-dose NTE administration group (300 mg/kg NTE dissolved in 1 mL distilled water orally), and high-dose NTE administration group (1000 mg/kg NTE dissolved in 1 mL distilled water orally). Dose was set based on our previous studies that showed the hypotensive effectiveness of plant extracts [19]. Blood pressure was measured before and at 1, 2, 4, 8, and 12 h after administration. As the measured blood pressure value was the lowest at 2 h after drug administration, the effects of repeated NTE administration on blood pressure in all groups of four rats each were evaluated using measurements obtained at this time point daily for 5 days.

### 2.6. Measurement of the Vasorelaxant Effect in Aortic Rings of SD Rat

The vasorelaxant effect was measured as described previously [20]. In brief, SD rats were anesthetized with urethane (1.2 g/kg, intraperitoneal injection). The aorta was cut into 2–3 mm length rings immediately. The rings were mounted in chambers filled with KH solution, subjected to resting tension of 1.0 g, and equilibrated for 50 min. To investigate the vasorelaxant effect of NTE, the rings were pre-constricted with phenylephrine (PE, 1 μM), and NTE (0.5, 1, 2, 5, or 10 μg/mL, dissolved in DMSO) was added cumulatively. Values of relaxation were expressed as the percentage of maximal constriction induced by PE.

To investigate the endothelial-cell-related vascular relaxation mechanism of NTE, the vascular relaxing effects of NTE on rings with or without endothelium were compared. Endothelial integrity was identified by the ability of acetylcholine (ACh, 10 μM) to induce the relaxation of vessels pre-constricted by PE (1 μM). Only the rings that induced relaxation higher than 85% were defined as endothelium-intact. Endothelium-removed rings were produced by mechanically removing endothelial cells with cotton swab. The rings that induced relaxation lower than 10% were defined as endothelium-removed.

To investigate whether vasorelaxation of NTE was related to the NO/cGMP pathway, the thoracic aortic rings were pretreated with NO synthase inhibitor (L-NAME, 100 μM), cyclooxygenase (COX) inhibitor (indomethacin, 10 μM), soluble guanylate cyclase (sGC) inhibitor (ODQ, 10 μM), or cGMP inhibitor (methylene blue, MB, 10 μM) for 20 min. The rings were pre-constricted with PE, and NTE was added at cumulative concentration.

To investigate whether vasorelaxation of NTE was related to K^+^ channels, thoracic aortic rings were pre-treated with the large-conductance Ca^2+^-activated K^+^ channel (BK_Ca_) blocker (TEA, 1 mM), voltage-activated K^+^ channel (K_V_) blocker (4-AP, 1 mM), ATP-sensitive K^+^ channel (K_ATP_) blocker (glibenclamide, 10 μM), or inward rectifier K^+^ channel (K_IR_) blocker (BaCl_2_, 10 μM) for 20 min. The rings were pre-constricted with PE, and NTE was added at cumulative concentration.

To investigate whether NTE vasorelaxation was related to Ca^2+^ channels, the experiments were carried out in Ca^2+^-free KH solution containing ethyleneglycol-bis(2-aminoethylether)-N,N‚N,’N′-tetraacetic acid (EGTA, 1 mM). Vasoconstriction was induced via the cumulative addition of CaCl_2_ (0.1, 0.3, 1, 3, or 10 mM) on aortic rings with or without pre-incubation of NTE (10 μg/mL) for 20 min, and with PE (1 μM) incubation for another 20 min.

To investigate whether NTE inhibits the action of angiotensin II (Ang II), vasoconstriction was induced by cumulative addition of Ang II (10^−9^, 10^−8^, 10^−7^, 10^−6^ M) on rings with or without pre-incubation of NTE (10 μg/mL) for 20 min.

### 2.7. Statistical Analysis

Statistical analyses were conducted using the GraphPad Prism 9 software (San Diego, CA, USA). Differences between groups were analyzed using multiple unpaired *t*-tests and two-way analysis of variance (ANOVA), followed by Bonferroni’s multiple comparison test. *p* < 0.05 was considered statistically significant.

## 3. Results

### 3.1. Acute Blood-Pressure-Lowering Effect of NTE

**Figure 1:**
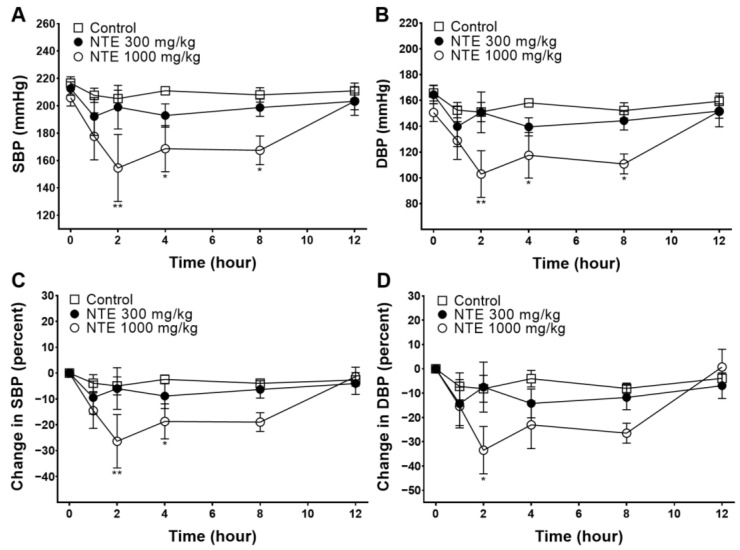
Systolic blood pressure (SBP) and diastolic blood pressure (DBP) values and percent changes via oral administration of 50% ethanolic extract of nutgall tree leaf (NTE) in spontaneously hypertensive rats (SHRs). (**A**) SBP, (**B**) DBP, (**C**) percent changes in SBP, (**D**) percent changes in DBP. Symbols and vertical lines indicate means ± SEM (*n* = 6). * *p* < 0.05, ** *p* < 0.01 vs. control.

Blood pressure values measured before and after the oral administration are shown in Figure 1A,B. SBP/DBP values of the NTE 1000 mg/kg group were 205.9 ± 6.1/150.6 ± 6.9 (0 h), 177.7 ± 17.2/128.9 ± 14.6 (1 h), 154.6 ± 24.5/102.9 ± 18.1 (2 h), 168.7 ± 16.9/117.5 ± 17.7 (4 h), 167.4 ± 10.5/110.8 ± 7.8 (8 h), and 203.4 ± 10.4/151.5 ± 12.0 (12 h) mmHg, and it significantly decreased at 2, 4, and 8 h after administration. Changes in blood pressure in each SHR were calculated and are shown in Figure 1C,D. Changes in SBP in the NTE 1000 mg/kg group showed significant differences 2 and 4 h after administration, and changes in DBP showed differences 2 h after administration. Changes in SBP and DBP were the lowest at 2 h (−26.4 ± 10.3% and −33.5 ± 9.8%, respectively). 

**Figure 2:**
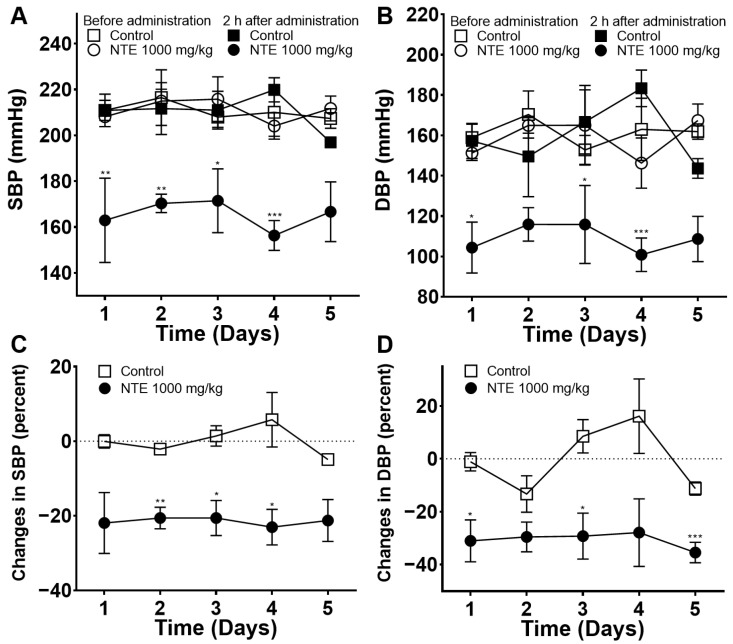
Systolic blood pressure (SBP) and diastolic blood pressure (DBP) values and percent changes via oral administration (1000 mg/kg) of 50% ethanolic extract of nutgall tree leaf (NTE) in spontaneously hypertensive rats (SHRs) for 5 days. (**A**) SBP, (**B**) DBP, (**C**) percent changes in SBP, (**D**) percent changes in DBP. Symbols and vertical lines indicate means ± SEM (*n* = 4). * *p* < 0.05, ** *p* < 0.01, *** *p* < 0.001 vs. control.

To determine the effect of daily administration of NTE on blood pressure, measurements of SBP and DBP were obtained 2 h after administering 1000 mg/kg of NTE daily for 5 days (Figure 2A,B). Changes in blood pressure in each SHR were calculated and are shown in Figure 2C,D. Both SBP and DBP showed significantly lower values than those in the control group on most days (average change: −21.5 ± 0.5% and −30.6 ± 1.5%, respectively).

### 3.2. Vasorelaxant Effect of NTE

**Figure 3:**
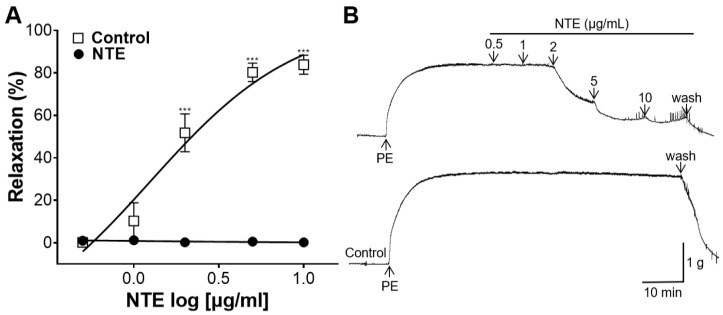
Vascular relaxation by 50% ethanolic extract of nutgall tree leaf (NTE) on aortic rings contracted with phenylephrine (PE, 1 μM). (**A**) Cumulative contraction curves and (**B**) representative traces. Symbols and vertical lines indicate means ± SEM (*n* = 5). *** *p* < 0.001 vs. control.

NTE showed a significant vascular relaxation on aortic rings compared to those in the control group (Figure 3). NTE showed the dose-dependent vasorelaxant effect, and the maximal effects were represented as 83.87 ± 4.48% at 10 μg/mL. 

### 3.3. Vasorelaxant Effect of NTE on Aortic Rings with or without Endothelium

**Figure 4:**
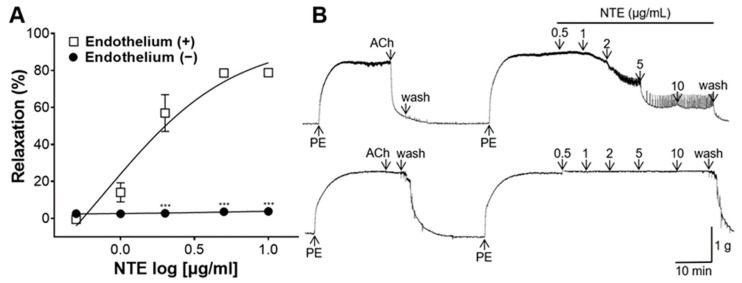
Vascular relaxation by 50% ethanolic extract of nutgall tree leaf (NTE) on aortic rings pre-constricted with phenylephrine (PE, 1 μM) depending on the presence of endothelium. (**A**) Cumulative contraction curves and (**B**) representative traces. Symbols and vertical lines indicate means ± SEM (*n* = 5). *** *p* < 0.001 vs. [Endothelium (+)].

NTE showed dose-dependent relaxing effect on endothelium-intact aortic rings, with a maximal effect of 78.80 ± 1.52% at 10 μg/mL; however, NTE showed little effect on endothelium-removed rings, with a maximal effect of 3.75 ± 1.04% at 10 μg/mL (Figure 4).

### 3.4. Effects of L-NAME or Indomethacin Pretreatment on NTE-Induced Vasorelaxation

**Figure 5:**
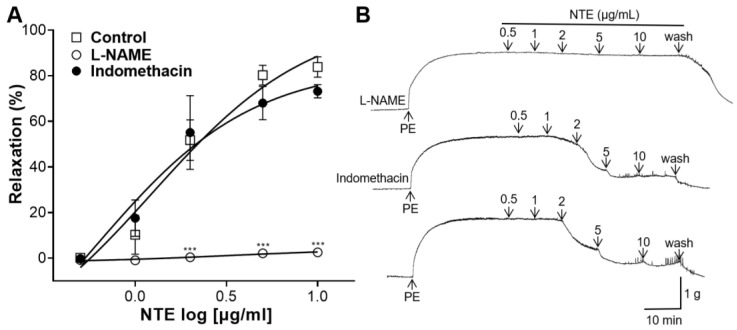
Effects of N^G^-nitro-L-arginine methyl ester (L-NAME, 100 μM) or indomethacin (10 μM) pretreatment on 50% ethanolic extract of nutgall tree leaf (NTE)-induced vasorelaxation. (**A**) Cumulative contraction curves and (**B**) representative traces. Symbols and vertical lines indicate means ± SEM (*n* = 4–5). *** *p* < 0.001 vs. control.

Since NTE showed endothelium-dependent vasorelaxant effects, its involvement in NO and COX mechanisms was investigated. L-NAME pretreatment significantly decreased the blood-vessel-relaxing effect of NTE; however, indomethacin pretreatment showed no significant difference from the control (Figure 5). 

### 3.5. Effects of ODQ or MB Pretreatment on NTE-Induced Vasorelaxation

**Figure 6:**
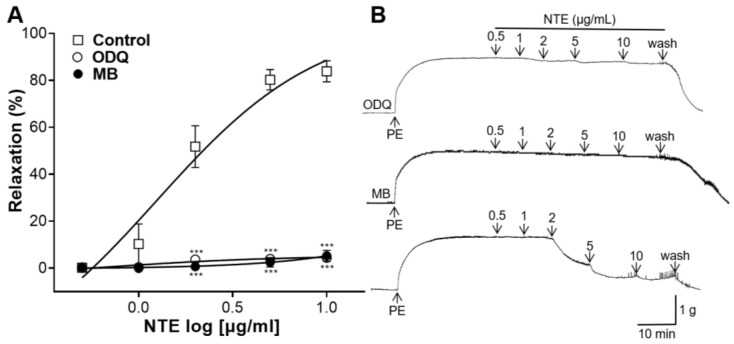
Effects of 1H-[1,2,4]oxadiazolo[4,3-a]quinoxalin-1-one (ODQ, 10 μM) or methylene blue (MB, 10 μM) pretreatment on 50% ethanolic extract of nutgall tree leaf (NTE)-induced vasorelaxation. (**A**) Cumulative contraction curves and (**B**) representative traces. Symbols and vertical lines indicate means ± SEM (*n* = 4–5). *** *p* < 0.001 vs. control.

The involvement of NTE in the NO-sGC-cGMP pathway was also investigated. Pretreatment with ODQ and MB significantly decreased the blood-vessel-relaxing effects of NTE (Figure 6).

### 3.6. Effects of K^+^ Channel Blockers Pretreatment on NTE-Induced Vasorelaxation

**Figure 7:**
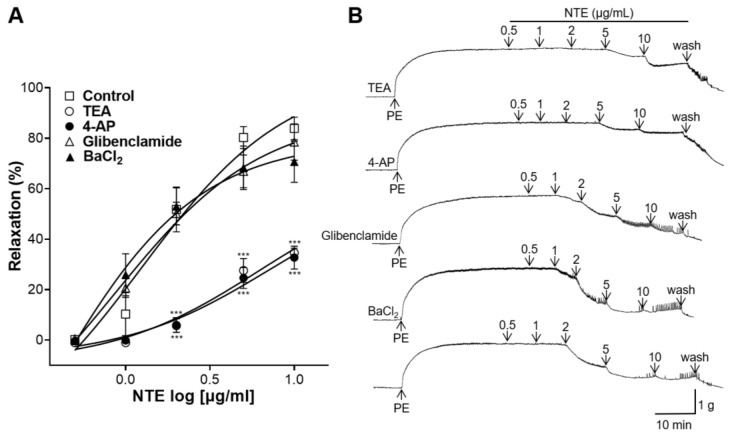
Effects of tetraethylammonium (TEA, 1 mM), 4-aminopyridine (4-AP, 1 mM), glibenclamide (10 μM), or barium chloride (BaCl_2_, 10 μM) pretreatment on 50% ethanolic extract of nutgall tree leaf (NTE)-induced vasorelaxation. (**A**) Cumulative contraction curves and (**B**) representative traces. Symbols and vertical lines indicate means ± SEM (*n* = 4–5). *** *p* < 0.001 vs. control.

K^+^ channel blockers were pretreated on aortic rings. TEA and 4-AP pretreatment significantly decreased the blood-vessel-relaxing effect of NTE; however, glibenclamide and BaCl_2_ pretreatment showed no significant differences compared with the control group (Figure 7).

### 3.7. Inhibitory Effect of NTE on Vasoconstrictive Action of Ca^2+^

**Figure 8:**
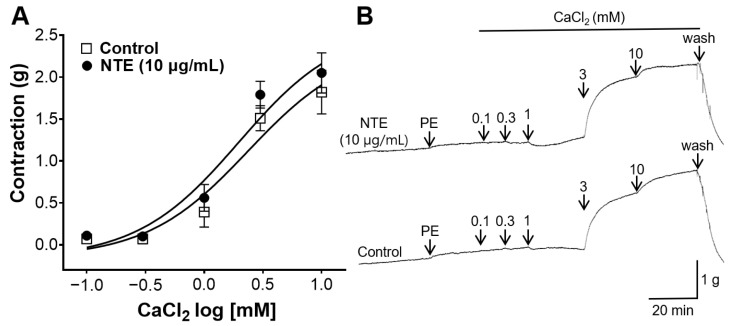
Inhibitory effect of 50% ethanolic extract of nutgall tree leaf (NTE) on vasoconstrictive action induced by CaCl_2_. (**A**) Changes in vascular tone via Ca^2+^-induced vasoconstriction and (**B**) representative traces. Symbols and vertical lines indicate means ± SEM (*n* = 5).

NTE was pretreated to the aortic rings in the chamber filled with Ca^2+^-free KH solution containing EGTA to investigate whether NTE blocks Ca^2+^ channels. NTE pretreatment showed no significant difference compared with the control group (Figure 8).

### 3.8. Inhibitory Effect of NTE on Vasoconstrictive Action of Ang II

**Figure 9:**
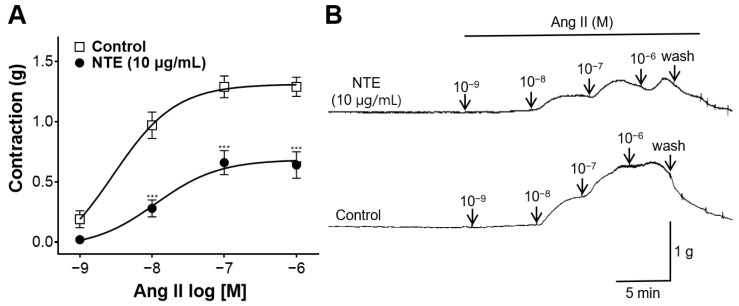
Inhibitory effect of 50% ethanolic extract of nutgall tree leaf (NTE) on vasoconstrictive action induced by angiotensin II (Ang II). (**A**) Changes in vascular tone via Ang II-induced vasoconstriction and (**B**) representative traces. Symbols and vertical lines indicate means ± SEM (*n* = 4–5). *** *p* < 0.001 vs. control.

NTE was pretreated to the aortic rings to investigate whether NTE inhibited the action of Ang II. NTE inhibited the constriction of rings induced by Ang II (Figure 9).

## 4. Discussion

The hypotensive effect of NTE was investigated in SHRs in this study. The SBP and DBP values of the NTE 1000 mg/kg group were significantly decreased at 2, 4, and 8 h after administration. In particular, changes in SBP and DBP were the lowest at 2 h (−26.4 ± 10.3% and −33.5 ± 9.8%, respectively). Moreover, to assess the effect of repeated administration of NTE, measurements were obtained 2 h after administering 1000 mg/kg of NTE daily for 5 days. The average changes in SBP and DBP were −21.5 ± 0.5% and −30.6 ± 1.5%, respectively, and no severe weight change or sudden death were observed during administration. SHRs develop hypertension with age, being pre-hypertensive for 6–8 weeks and then hypertensive over 12–14 weeks [21]. In our experiment, we used 18-week-old SHRs that had already developed hypertension; our results thus showed that NTE had a blood-pressure-lowering effect in hypertensive individuals. Because a decrease in SBP reduces the risk of CVDs, NTE appears to serve as a healthy functional food that reduces cardiovascular risk [5]. Previous experiments have revealed the results of acute toxicity tests using NT fruits (LD_50_ > 5000 mg/kg); however, data on toxicity tests using NTE remain lacking [22]. Therefore, research on the effectiveness and toxicity of the long-term administration of NTE is needed.

Vascular tone mainly determines blood flow resistance, and thus plays a major role in regulating blood pressure [23]. Therefore, the blood-vessel-relaxing effect of NTE and its possible mechanism of action were studied. NTE showed a dose-dependent vasorelaxant effect, and the maximum value was 83.87 ± 4.48% at 10 μg/mL. The endothelium is responsible for controlling the tone of vascular smooth muscle cells (VSMC) and the resistance of blood vessels through the synthesis of vasodilator substances, including NO and prostacyclin (PGI_2_) [24]. NTE showed a dose-dependent relaxing effect on aortic rings with the presence of endothelium, with the maximum effect of 78.80 ± 1.52% at 10 μg/mL; however, NTE showed little vasorelaxant effect on endothelium-removed aortic rings, with a maximal effect of 3.75 ± 1.04% at 10 μg/mL. Therefore, our results suggest that NTE induces vasorelaxation via an endothelium-dependent pathway.

NO produced by NO synthase in endothelial cells diffuses into adjacent VSMC and binds to sGC to synthesize cGMP [25]. cGMP reduces Ca^2+^ release from the sarcoplasmic reticulum and tension in VSMC [26]. In addition, endothelial cells release PGI_2_, which is synthesized by COX [27]. PGI_2_ binds to VSMC receptors and activates adenylate cyclase to synthesize cyclic adenosine monophosphate [25]. This study demonstrated that the vascular relaxing effect of NTE was decreased in rings pre-incubated with L-NAME (NO synthase inhibitor), but no significant decrease was observed in rings pre-incubated with indomethacin (COX inhibitor). This finding indicated that vasorelaxation of the NTE was related to NO. Furthermore, rings incubated with ODQ (sGC inhibitor) or MB (cGMP inhibitor) showed a reduction in the vasorelaxant effect of NTE. These results suggest that NTE induces vasorelaxation via the NO-sGC-cGMP pathway.

K^+^ channels are the main ion-conductive pathways in VSMCs, and activation of K^+^ channels regulate the membrane potential, resulting in vasodilation [28]. Regarding K^+^ channels, four different types of K^+^ channels (BK_Ca_, K_V_, K_ATP_, and K_IR_) are expressed in VSMCs, and the most prevalent K^+^ channels are BK_Ca_ and K_v_ channels [23,29]. The dysfunction of these K^+^ channels is correlated with increased vascular tone in patients with hypertension [29]. This study demonstrated that the vascular relaxing effect of NTE was reduced in rings pre-incubated with TEA (BK_Ca_ blocker) and 4-AP (K_V_ blocker). However, glibenclamide (K_ATP_ blocker) and BaCl_2_ (K_IR_ blocker) pretreatment showed no significant difference from the control. Therefore, our study suggests that NTE relaxes blood vessels by activating the BK_Ca_ and K_V_ channels.

VSMC maintain proper vascular function and regulate blood flow, and Ca^2+^ plays a pivotal role in these processes [30]. Concentrations of Ca^2+^ are changed by Ca^2+^ influx through Ca^2+^-permeable channels and Ca^2+^ release from intracellular stores [30]. In our study, we investigated whether Ca^2+^ channels were involved in the vascular relaxation mechanism of NTE, and our results showed that NTE pretreatment caused no significant decrease in the tone of aortic rings that are induced vasoconstriction by Ca^2+^. Therefore, our results indicate that the vascular relaxing effect of NTE was irrelevant with Ca^2+^ channels.

Another important factor controlling vascular tone and blood pressure is Ang II, a key component of the renin–angiotensin system [31]. Ang II modulates vasomotor tone through its potent vasoconstrictive effects [32]. The binding of Ang II to AT_1_ receptor, which is distributed in the VSMC of most arteries at low-to-moderate densities and in endothelial cells, induces an increase in intracellular Ca^2+^, leading to the contraction of VSMC [33,34]. In this study, we investigated whether NTE reduces the vasoconstrictive effect of Ang II and found that NTE inhibits the constriction of rings induced by Ang II. Ang II is involved in vasoconstriction, and excessive Ang II is thought to be associated with impaired endothelial function and increased cardiovascular risk [31,35]. Therefore, our results indicate that NTE inhibits the action of Ang II and can help reduce associated cardiovascular risks.

Functional foods, especially those from plant sources containing biologically active compounds, have the potential to protect against degenerative diseases, including CVDs [36]. Various bioactive compounds from plants exhibit vasorelaxant effects through multiple mechanisms such as the NO-sGC-cGMP pathway, eicosanoid system, and K^+^ and Ca^2+^ channels [37]. Our research also shows that NTE can be applied to high blood pressure through various mechanisms, making it the basis for research that can be developed into a functional food for cardiovascular health. However, because the components of NTE were not analyzed, further research is needed to identify the active components of NTE for the development of functional foods. In addition, we conducted experiments using the leaf of NT in this study; however, it has been revealed that not only leaf but also fruit, stem, and *Galla chinensis* have various ingredients and effects [13]. Therefore, further studies on various parts of the NT are required.

## 5. Conclusions

NTE showed an acute blood-pressure-lowering effect in SHRs; in particular, the lowest blood pressure was observed 2 h after administration. Daily NTE administration for 5 days also resulted in a similar blood-pressure-lowering effect. In addition, NTE has an endothelium-dependent relaxing effect on the rat thoracic aorta. The vasorelaxant effect of NTE involves the NO-sGC-cGMP pathway, opening of BK_Ca_ and K_V_ channels, and a reduction in the vasoconstrictive action of Ang II. Therefore, our experimental results provide basic data indicating that NTE can be used as a functional food for cardiovascular health.

## Figures and Tables

**Table 1:** Bioactive compounds of 50% ethanolic extract of nutgall tree leaf (NTE).

Total Phenolic	Total Flavonoid	Total Anthocyanin
187.04 ± 2.69	90.20 ± 0.59	1.30 ± 0.29

Values are expressed as means ± SD (*n* = 3).

## Data Availability

The original contributions presented in the study are included in the article, further inquiries can be directed to the corresponding author.
